# Morphological prediction of glaucoma by quantitative analyses of ocular shape and volume using 3-dimensional T2-weighted MR images

**DOI:** 10.1038/s41598-019-51611-0

**Published:** 2019-10-22

**Authors:** Yasuko Tatewaki, Tatsushi Mutoh, Kazuko Omodaka, Benjamin Thyreau, Izumi Matsudaira, Hiroaki Furukawa, Keiji Yamada, Keiko Kunitoki, Ryuta Kawashima, Toru Nakazawa, Yasuyuki Taki

**Affiliations:** 1 Department of Nuclear Medicine and Radiology, Institute of Development, Aging and Cancer, Tohoku University, Sendai, Japan; 2 Department of Geriatric Medicine and Neuroimaging, Tohoku University Hospital, Sendai, Japan; 3 Department of Ophthalmology, Tohoku University Graduate School of Medicine, Sendai, Japan; 4 Smart-Aging Research Center, Tohoku University, Sendai, Japan; 5 Department of Functional Brain Imaging, Institute of Development, Aging and Cancer, Tohoku University, Sendai, Japan

**Keywords:** Glaucoma, Retinal diseases, Retina

## Abstract

Elongated axial length of the eye increases the morbidity of glaucoma. Myopia also associates with elongated axial length, and such ellipsoid shape of the eyeball strongly contributes its pathogenesis. Morphological features of the eyeballs, which could be important factors for developing glaucoma, have not been well described. The aim of this study was to investigate the three-dimensional (3D) topographic features of glaucomatous eyeballs with/without myopia to evaluate the potential of those features for predicting glaucoma. Using a 3.0-tesla MRI, volume-isotropic turbo-spin-echo acquisition T2-weighted images were obtained from 55 patients with glaucoma and 22 controls to delineate the eyeballs. Eyeball volumes, axial lengths and transverse lengths were semi-automatically calculated and compared between four groups: normal, myopia, glaucoma, and glaucoma with myopia. Both glaucoma and myopia increased the eyeball volume compared to the normal eyes. An increased anisotropy ratio (axial/transversus length) was observed in myopic eyes compared to normal, whereas in the glaucomatous eyes, with or without myopia, no increase in anisotropy ratio was observed. Increasing volume of eyes can be caused by myopia and glaucoma. Myopic eyes were ellipsoid in shape, but there was less anisotropy and a near-spherical shape in glaucomatous eyes, even in glaucomatous myopic eyes.

## Introduction

Glaucoma is the leading cause of blindness worldwide, and the number of patients is still growing^1^, but its underlying mechanism remains unclear. Previous studies have revealed several risk factors, including: advanced age, family history, high intraocular pressure (IOP), and increased cup-to-disk ratio or its asymmetry on funduscopy^2^. Furthermore, elongated axial length of the eye has been implicated to increase the morbidity of glaucoma^3–6^. Given these observations, morphological features of the eyeball, including the retrobulbar structure in the orbit, could be important factors contributing to the development of glaucoma. However, routine ophthalmological examinations (e.g., stereoscopic funduscopy and B-mode echography for the assessment of the anterior chamber, lens, fundus, optic-nerve head and axial-length abnormalities) currently provide only limited morphological information in the transaxial and vertical dimensions of the eyeball.

Magnetic resonance imaging (MRI) is an appropriate device for elucidating such inaccessible information noninvasively. Application of a high-resolution 3-dimensional (3D) MRI technique, coupled with the development of image analysis, is beneficial for imaging the volume and shape of the whole eye precisely and quantitatively. Yet, the topographic features of whole eyes have not been described extensively for glaucomatous eyes, although some topographic features of myopic eyes have been evaluated with MRI^7,8^. Myopic eyes tend to form a sphero-elliptic shape, with trends toward increases in the axial and vertical dimensions^9,10^. Considering the aforementioned findings on myopia and glaucoma, we hypothesized that an understanding of the morphological aspects of glaucomatous eyes would provide crucial information about the etiology of glaucoma, and the feasibility of predicting it, as well as indicating whether there is a causal relationship between glaucoma and myopia. The aim of this study was to investigate the topographic features of whole eyeballs with glaucoma and/or myopia, using high-resolution 3D T2-weighted images, with anatomical image-segmentation for precise geometric and volumetric analyses and to evaluate the potential of those features for predicting glaucoma.

## Results

### Correlations between whole-eye morphological parameters and demographic data

**Figure 1:**
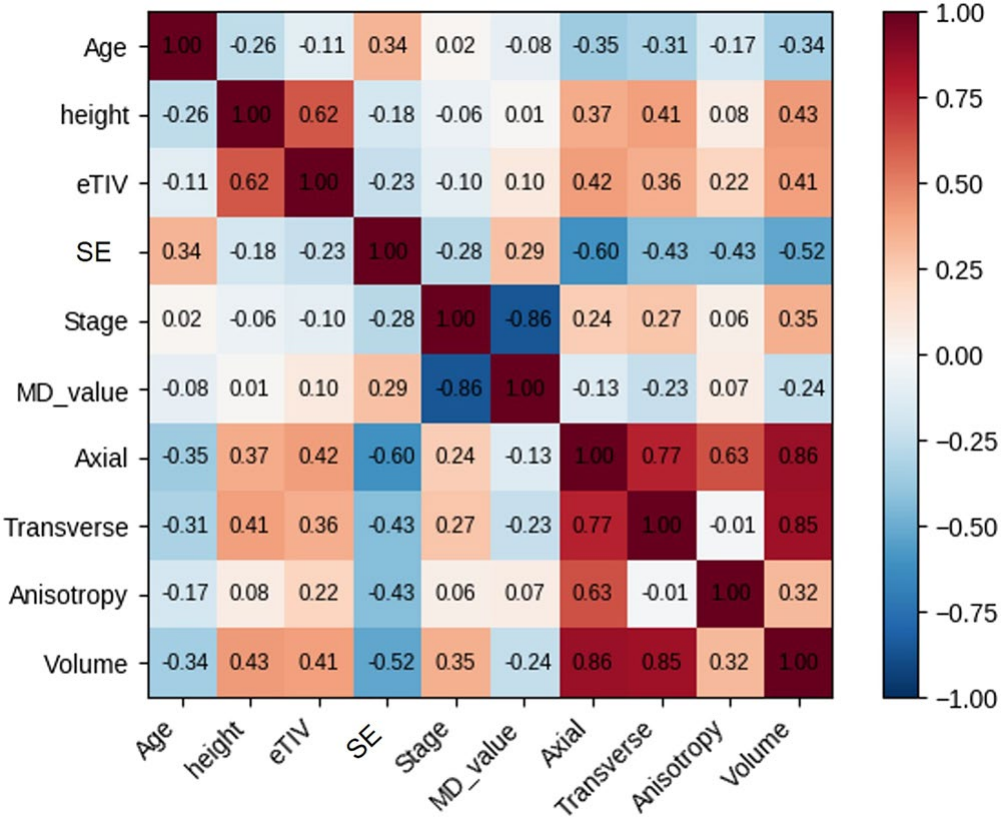
Correlations between the morphological parameters of whole eyes and the demographic data. Correlation coefficients (p) between pairs of variables among the whole-eye morphological parameters and the demographic data. eTIV = Estimated total intracranial volume; SE = Spherical equivalent refraction; Stage = Clinical glaucoma stage; MD = Mean deviation; SD = Standard deviation; Anisotropy = Anisotropy ratio (axial length/ transverse length); Volume = Eyeball volume.

The results of Spearman-correlation analyses are shown in Fig. 1. Across all eyes, the eyeball volumes were: strongly correlated with axial length (p = 0.84, *P* < 0.0001) and transverse length (p = 0.83, *P* < 0.0001); moderately correlated with height (p = 0.40, *P* < 0.0001), eTIV (p = 0.40, *P* < 0.0001) and SE (p = −0.57, *P* < 0.0001)); and mildly negatively correlated with MD (p = −0.32, *P* = 0.0036) and age (p = −0.39, *P* = 0.001). The eye anisotropy ratio (axial length/transverse length) showed a strong positive correlation with axial length (p = 0.63, p < 0.0001), and a moderate negative correlation with SE (p = −0.41, p < 0.0001) but no statistically significant correlations with the other demographic data.

To evaluate an effect of glaucoma on the morphological parameters, stratified analyses were performed on the data from the normal and non-myopic glaucoma groups (i.e., the two non-myopic groups). In those analyses, there was a mild negative correlation between eye volume and MD (p = −0.39, *P* = 0.0025), but no correlation between the anisotropy ratio and MD. Excluding participants with PPG, the glaucoma stage was also significantly correlated with eyeball volume (p = 0.36, *P* < 0.0001), axial length (p = 0.26, *P* = 0.0052), and transverse length (p = 0.31, *P* = 0.0006), but there was no correlation with the anisotropy ratio (p = 0.03, *P* = 0.12).

### Visual inspection of 3D-rendering average eyes

**Figure 2:**
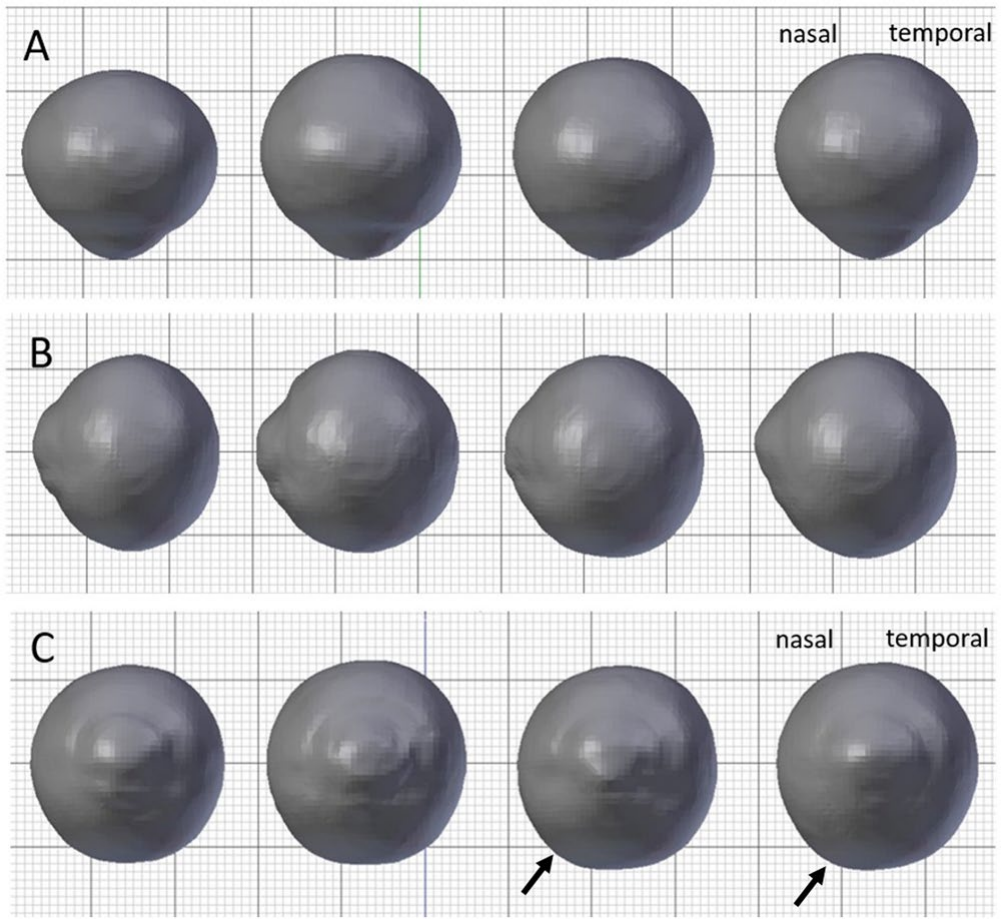
Averaged eye morphology of each group. The columns, from left to right, show the average eye morphology associated with (1) normal/non-myopia (control), (2) normal/myopia, (3) glaucoma/non-myopia and (4) glaucoma/myopia. (**A**) Superior view, (**B**) Lateral view (Temporal side), (**C**) Anterior view. The myopic eye shows an elongated axial length and ellipsoid shape, as compared with the control eye. The glaucomatous eyes show enlargement, but less anisotropy and near-spherical patterns with slightly asymmetrical protrusion in the infero-nasal direction (black arrows), even in glaucomatous-myopic eyes.

3D-rendering models demonstrated representative characteristics of whole-eye morphometry associated with the four groups, as shown in Fig. 2. Compared with normal, the average myopic eye was bigger, ellipsoidal with increased retrobulbar curvature. In contrast, glaucomatous eyes—with or without myopia, tended to be bigger than normal and spherical, and tended to protrude slightly in the infero-nasal direction.

### Group-comparisons of morphological parameters of the eye

**Figure 3:**
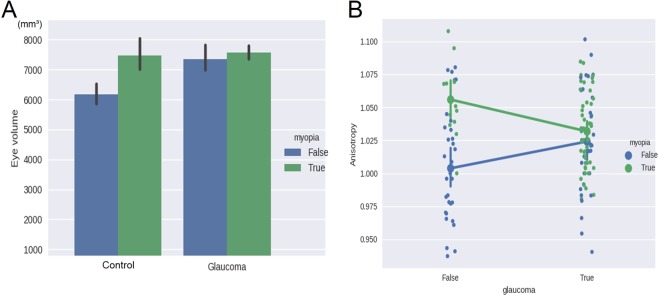
Results of group comparisons. Mean and SD values of eye volume (**A**) and anisotropy ratio (axial length/ transverse length) (**B**) in each group. (**A**) Both factors, myopia and glaucoma, had a statistically significant effect on eyeball volume, increasing it relative to that of the normal controls. No interaction effect on eyeball volume was observed between myopia and glaucoma. (**B**) Increased anisotropy (ellipsoid shape) was observed in both non-glaucomatous and glaucomatous myopic eyes, versus normal control. There was an interaction effect between glaucoma and myopia: compared to myopic eyes without glaucoma, a significant reduction in the anisotropy ratio was recorded in eyes with glaucomatous myopia.

Characteristics of the eyes in each group are listed in Table 1. Both myopia and glaucoma tended to increase ocular volume relative to the normal eyes (myopia versus control, *P* = 0.003; glaucoma versus control, *P* = 0.007; glaucoma/myopia versus control, *P* < 0.001), but no statistically significant interactions were observed between the two disease conditions (Fig. 3A).

**Table 1:** Whole-eye morphological parameters and demographic data.

	Normal control	Myopia	Glaucoma	Glaucoma with Myopia	PPG
Number of eyes	30	13	33	51	27
IOP (mmHg) (mean ± SD)	15.1 ± 2.6	13.3 ± 1.9	12.8 ± 2.6^‡^	12.7 ± 2.1^§^	14.0 ± 2.8
Gender (Men/Women)	12/18	8/5	11/22	17/32	12/15
Age (years), (mean ± SD)	59.7 ± 9.5	48.0 ± 7.0^‡^	57.4 ± 9.7	54.2 ± 8.9	57.9 ± 9.3
Height (cm), (mean ± SD)	160.2 ± 7.0	168.6 ± 5.9^‡^	160.3 ± 10.0	162.4 ± 8.5	161.0 ± 7.0
eTIV (mm^3^ × 10^3^) (mean ± SD)	1383.8 ± 143.3	1506.2 ± 131.9^*^	1364.2 ± 125.7	1397.1 ± 99.0	1413.7 ± 122.8
SE (D), mean (SD)	−0.39 ± 1.46	−5.3 ± 1.78^§^	−0.97 ± 1.03	−5.58 ± 1.84^§^	−1.88 ± 2.65^*^
Visual sensitivity (MD) (mean ± SD)	0.54 ± 1.0	−0.05 ± 0.86	−10.20 ± 7.3^§^	−10.04 ± 8.0^§^	−0.25 ± 1.5^†^
Eye volume (mm³) (mean ± SD)	6182.3 ± 909.2	7467.6 ± 1028.6^‡^	7357.3 ± 1265.7^§^	7558.9 ± 843.8^§^	6888.4 ± 850.8
Axial length (mm) (mean ± SD)	23.2 ± 1.3	25.6 ± 1.5^§^	24.6 ± 1.8^‡^	25.2 ± 1.3^§^	24.4 ± 1.5^*^
Transverse length (mm) (mean ± SD)	23.2 ± 0.8	24.3 ± 1.4^*^	24.2 ± 1.4^†^	24.4 ± 1.1^§^	23.4 ± 11.3
Anisotropic ratio, (mean ± SD)	1.00 ± 0.04	1.06 ± 0.03^‡^	1.02 ± 0.05	1.04 ± 0.03^*^	1.02 ± 0.05

PPG = Preperimetric glaucoma; IOP = Intraocular pressure; eTIV = estimated Total Intracranial Volume; SE = Spherical equivalent refraction; D = Diopters; MD = Mean deviation; SD = Standard deviation; Anisotropic ratio = axial length/transverse length.

Significance, by Dunnett’s multiple-comparison test: ^*^P < 0.05; ^†^P < 0.01; ^‡^P < 0.005; ^§^P < 0.001.

However, there was the interaction effect of myopia and glaucoma on the anisotropy ratio (*P* = 0.04). In the non-glaucomatous eyes, a significant ellipsoid shape was observed in myopic eyes compared to normal eyes (*P* = 0.0017), whereas in the glaucomatous eyes, no significant ellipsoid feature was observed in myopic eyes compared to non-myopic eyes (*P* = 0.39). Conversely, compared with myopic eyes without glaucoma, a significant reduction in the anisotropy ratio was recorded in eyes with glaucomatous myopia (*P* = 0.0078, Fig. 3B).

### Diagnostic potential of morphological parameters for distinguishing between glaucoma, PPG and myopia

**Figure 4:**
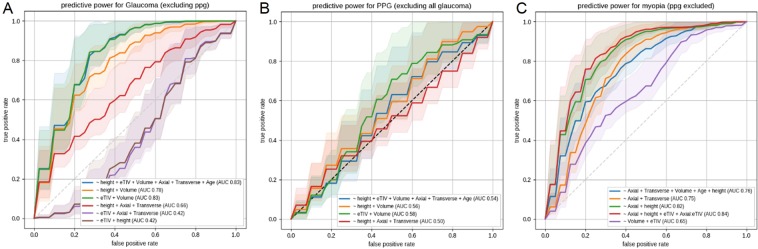
Predictive power of morphological parameters for glaucoma or myopia. Receiver operating characteristic curves and their Area under the curve (AUC) indicate predictive power for (**A**) Glaucoma (excluding Preperimetric glaucoma (PPG)) versus non-glaucoma, (**B**) PPG vs non-glaucoma, (**C**) Myopia (Spherical equivalent refraction ≤−3) versus non-myopia. AUC values are computed on the average curve (the actual line), and the shaded area depicts one-half-SD around the curve. Curves that go below the diagonal indicate that the model was overfitting.

To distinguish between glaucomatous and control eyes (Fig. 4A), the most-predictive model had two parameters, eyeball volume and eTIV, and reached an Area under the curve (AUC) of 0.83. At a sensitivity of 80%, the model produced 25% false-positives and, if 50% false-positives could be tolerated, the specificity would be close to 1.

For discriminating PPG eyes from control eyes, the best-performing model reached an AUC of 0.58, a relatively low, yet significant, value (Fig. 4B). Notably, that model used the same predictor variables as did the best model for glaucoma detection.

For myopia prediction, a model using the eye axial and transverse lengths reached an AUC of 0.78, but the eyeball volume was not a better predictor (AUC = 0.69). The prediction could be made more accurate (AUC = 0.83) by using a general size-normalization factor (i.e., height) instead of the transverse length, whereas adding further variables, such as an interaction term of axial length and brain volume, resulted in only marginal improvement. At a sensitivity of 80%, the best model produced 29% false-positives (Fig. 4C).

## Discussion

The topographical details associated with glaucoma, including volume and shape of the eye, remain unclear. In this study, we reported the feasibility of determining the characteristic shape of glaucomatous eyes using 3D high-resolution MRI, with quantitative analysis. Our results suggest a new insight: that increasing ocular volume can be caused by both myopia and glaucoma. Myopic eyes, even excluding those with severe or pathological myopia, were characterized by elongated axial length and ellipsoid shape, but glaucoma-related eye enlargement was characterized by less anisotropy and a near-spherical shape, even in glaucomatous myopic eyes.

We found a correlation between eyeball volume and severity of glaucoma after excluding the effect of myopia. This result, that large eyeball volume might reflect glaucomatous change, confirms the previous descriptions of elongated axial length being associated with increased glaucoma morbidity^3,4^. It also indicates that morphological features of the eye could be risk factors for developing glaucoma. In particular, the anisotropy ratio could provide specific information about ophthalmological conditions. In our results, myopic eyes showed a more ellipsoidal shape, with elongated axial length, which is consistent with previous reports using 2D or 3D MRI analysis of eyes^8,10^. However, the glaucomatous eyes in our study demonstrated preservation of a near-spherical shape with mild protrusion in the infero-nasal direction, despite eye volume being markedly enlarged with increasing glaucoma severity.

This is the first description of this 3D feature of glaucomatous eyes *in vivo*. Spherical deformation of glaucomatous eyes may result from increasing IOP or turbulent flow in the orbit. Previously, during animal experiments in a glaucoma model, increasing the IOP deformed flattened chick eyes into near-spherical enlargement^11–13^, which appears compatible with our results. Coudriller *et al*.^14^ previously reported that the meridional strain response in the peripapillary sclera was significantly lower and stiffer for glaucoma specimens than for non-glaucoma specimens, which might indicate excessive extension of the peripapillary sclera in the meridian direction consequent to spherical enlargement of glaucomatous eyes. Such variability of eyeball deformation could be an important clue to understanding the pathogenesis of glaucoma and etiological differences between myopia and glaucoma.

Deformation of the eyes due to myopia and that due to glaucoma may be definitively different regarding their periods of onset and their pathogenesis. With myopic eyes, the axial length elongates during early adulthood, and posterior staphyloma develops during early middle age. Such ellipsoid deformation may be attributed to both genetic and environmental factors, whereas the structural change associated with glaucoma mainly appears in the elderly, possibly as a senile change. Several studies have shown that corneas and the lamina cribrosa/posterior sclera in older adults are more rigid and less elastic than those in younger individuals^15,16^. Such variability in the deformation of eyeballs could explain the pathogenesis of glaucoma. In this study, spherically enlarging eyes with mid-protrusion in the posterior fundus could be correlated with glaucoma severity. This deformation pattern is accompanied by a retrobulbar structure stretching almost equally in the meridian direction from the optic nerve head. Consequently, excessive tension and distortion of retinal nerve fibers and retinal perfusion insufficiency could lead to a progressive visual field defect. Furthermore, spherical enlargement added by bumps in the inferonasal portion of the posterior fundus could facilitate vulnerability of glaucoma throughout irregular stretching of retinal nerve fibers and the lamina cribrosa. In contrast, the ellipsoid shape of purely myopic eyes accompanied by enlargement predominantly of the equatorial portion of the a prolate (elongated), spheroid-like eyeballs, but stretching and distortion of the posterior fundus, seems to be less than that of eyes with the spherical enlargement pattern. Such imbalanced eyeball deformation that depends on directions could have a preservation effect on retinal nerve fibers and optic nerve heads and might be associated with decreased glaucoma-related morbidity. Hence, we speculate that spherical deformation may cause glaucomatous optic neuropathy via excessive stretching of the posterior fundus, whereas ellipsoid deformation may not. In this context, anisotropic eyes would have some tolerance for glaucomatous damage. Nitta *et al*.^17^ suggest that the rate of the progression of visual field defects due to glaucoma was significantly slower in the highly myopic group with marked elongated axial length compared with that in the non-myopic group. Their report suggests the possibility that ellipsoid eyes associated with myopia can preserve retinal nerve fibers from glaucomatous optic neuropathy, which supports our results. Our results, however, are discrepant from those in numerous reports that found that the presence of myopia increased the morbidity associated with glaucoma^3–6^. In those reports, only axial length and refractive errors were considered variables associated with myopia. Morphological information of whole eyes (including the transverse length and ellipsoid ratio) were not mentioned. Our results showed that spherical, large eyes with myopic-glaucoma exhibited elongation of the axial length. Significant proportion of participants with myopia in those previous studies might have large, spherical eyes, not ellipsoid eyes presumably associated with glaucoma. Thus, the deformation pattern of the posterior fundus in the meridian direction might be an important clue to the pathogenesis of glaucoma. Furthermore, myopia-affected anisotropic eyes during middle age might be predisposed to deform spherically more easily during the gerontic period, compared with non-myopic eyes, and could develop glaucoma. Further longitudinal investigations, including information on whole-eye morphology, are needed to elucidate causal relations between myopia and glaucoma.

Our ROC analysis with logistic regressions suggested the novel possibility that this non-invasive MRI method could independently identify patients with glaucoma who are otherwise not conscious of their visual field deficit. The best model for predicting glaucoma was simply associated with eyeball volume and eTIV. The eyeball volume was a significantly better predictor than the eye axial or transverse length, and this effect was most apparent when individual brain size was controlled for. The fact that eTIV is better than height as a control for normalization of eyeball volume can be explained by the fact that eTIV is a cubic scale, like volume, whereas height is a one-dimensional scale. Interestingly, the best model for predicting PPG was the same as that for predicting glaucoma. Although the predictive value was too low to be useful as a diagnostic test for PPG, it may be acceptable as a screening index. Additionally, our statistical analysis confirmed that the anisotropy of the eye is a good predictor for myopia and we observed that the effect it is mostly driven by variance in the axial component, while the transverse component may be acting as per-subject normalization.

This study has some limitations. First, we analyzed only cross-sectional data, and longitudinal studies are needed to confirm a causal effect between characteristics of eye geometry and glaucoma. Second, we used a specific MRI sequence, T2-VISTA, and semi-automatic image-analysis method for delineating precise whole-eye morphology, which could be unsuitable for a larger study or general applicability. The coverage of our T2-VISTA image was only from the orbit to the optic chiasm. Thus, more commonly available 3D MRI sequences, such as MPRAGE, covering the whole brain as well as the orbit, would be beneficial for large-scale use. Finally, our segmentation method was time-consuming, and involved a great deal of careful attention, to provide consistent results. Based on the magnitude of the effect size that we found (hundreds of mm^3)^ in the morphological parameters, we have determined that the use of a high-resolution image, with semi-automatically manual segmentation, could, in principle, be avoided. In fact, we have already started developing assessment system, relying only on a T1-weighted sequence and a fully-automated analysis, to broaden the applicability for the analysis of large cohorts of routine MRIs.

To conclude, this study clarified the clinical importance of 3D-geometrical information about the eye, for predicting glaucoma: When large eyes are observed on MRI, there is reason to be concerned about possible glaucoma, as well as myopia. Specifically, axial-elongated ellipsoid eyes indicate the possibility of myopia, whereas nearly spherical larger eyes indicate the possibility of glaucoma. Glaucoma is difficult to diagnose during the early stage because patients have few or no complaints about visual field defects at that stage, leading to delayed diagnoses and initiation of treatment. Existing ophthalmological methods, including OCT and visual field testing, are usually applied only when there are subjective complaints about the eyes. MRI is broadly available in Japan, even for individuals with no eye complaints. It is performed for brain medical checkups because of its repeatability and noninvasive nature. Our results suggest that brain MRI could be a superior technique for detecting the presence of glaucoma at a comparatively early stage, especially when MRI is being used for brain checkups with no subjective complaints about the patients’ eyes. Therefore, this image-driven approach for glaucoma detection could contribute to reducing the high prevalence of glaucoma in today’s aging society.

## Materials and Methods

### Subjects

This cross-sectional study was conducted at Tohoku University and involved 77 participants. There were 55 patients with glaucoma or preperimetric glaucoma (PPG; see the “*Ophthalmological examinations*” section) (ages: 56.2 ± 10.3 years; range: 40–75) and 22 visually normal controls (ages: 56.0 ± 9.3 years; range: 41–78). There were no significant differences in age or height between the patients with glaucoma and the control groups. The inclusion criteria for the glaucoma group were: 1) diagnosis of open angle glaucoma (OAG), including either primary OAG or normal-tension glaucoma; and 2) a glaucomatous visual field in accordance with the Anderson-Patella classification^18^ (see below). The exclusion criteria for all participants were: (1) ocular disease other than OAG; (2) no high myopia (which was defined as a spherical equivalent refractive error worse than −8.00 diopters); (3) systemic disease affecting the visual field; (4) history of intraocular surgery; and (5) cataracts greater than grade 1 of the Emery-Little classification^19^. This study was approved by the Ethics Committee of Tohoku University Graduate School of Medicine (2015-1-560). All experiments were performed in accordance with the Declaration of Helsinki. Written informed consent was obtained from all participants.

### Ophthalmological examinations

Participants first underwent a fundus examination. Mean deviation (MD), which is a quantitative index representing visual sensitivity, was measured with the Swedish interactive threshold algorithm-standard strategy of the 24-2 program on the Humphrey Visual Field Analyzer (Carl Zeiss Meditec)^20^. The MD values from fields considered reliable (<20% fixation errors, <15% false positives and <33% false negatives) were used. A glaucomatous visual field was defined, according to the Anderson-Patella criteria^18^, by one or more of the following: (1) a cluster of three points with probabilities of <5% on the pattern deviation map in at least one hemifield (including ≥1 point with probability of <1% or a cluster of two points with a probability of <1%); (2) glaucomatous hemifield test results outside the normal limits; or (3) a pattern standard deviation beyond 95% of normal limits, as confirmed on at least 2 reliable examinations. PPG was defined based on evidence of structural changes such as disk cupping, rim notching, or retinal nerve fiber layer defects, with no evidence of any glaucomatous visual field defects.

Refraction was determined with an SRW-5000 auto refractor (Shin-Nippon). Myopia was defined as a spherical equivalent refraction (SE) of worse than −3.00 diopters. Next, the 154 eyes were classified into 5 groups: (1) normal/non-myopia (control), (2) normal/myopia, (3) glaucoma/non-myopia and 4) glaucoma/myopia. The numbers of eyes/group were: 30 (participant ages: 59.7 ± 9.5 years; SE: −0.39 ± 1.5 D), 13 (ages: 48.0 ± 7.0 years; SE: −5.3 ± 1.8 D), 33 (ages: 57.4 ± 9.7 years; SE: −0.97 ± 1.0 D) and 51 (ages: 54.2 ± 8.9 years; SE: −5.58 ± 1.8 D), respectively. Separately, 27 eyes were categorized as a single group of PPG with or without myopia (ages: 57.9 ± 9.3 years; refraction: −1.88 ± 2.7 D) (Table 1). We did not distinguish between right and left eyes, there were no significant differences in demographic parameters based on that factor.

### MRI acquisition

MRI data were acquired using a 3.0-tesla Achieva scanner (Philips) equipped with an eight-element head-coil. High-resolution 3D isotropic T2-weighted images were used to delineate the morphological features of the participants’ eyes, using the volume-isotropic turbo-spin-echo acquisition (VISTA) sequence, with these imaging parameters: Repetition time (TR) = 2500 ms, Echo time (TE) = 325 ms, flip angle = 90°, slices thickness = 0.7 mm, field of view = 241 × 241 × 87 mm^3^, acquisition matrix size = 352 × 352, reconstructed voxel size = 0.7 × 0.7 × 0.7 mm^3^; scan duration = 5 min, 20 s. To avoid motion artifacts caused by eye movements, participants were instructed to fixate on a map projected on a monitor in the MRI scanner during data acquisition. We also acquired a general-purpose structural image of each head, using the 3D magnetization-prepared rapid acquisition gradient echo (MPRAGE) sequence, with these parameters: TR = 8.70 ms, TE = 3.1 ms, flip angle = 8°, slices thickness = 0.7 mm, field of view = 256 × 256 × 256 mm^3^, acquisition matrix size = 365 × 365, reconstructed voxel size = 0.7 × 0.7 × 0.7 mm^3^; scan duration = 5 min, 19 s.

### Measurement of intracranial volume

To control for head size, we computed the brain volume of each participant as a normalization coefficient. For each subject, the MPRAGE image was processed with the automatic software “*hippodeep”* (available at https://github.com/bthyreau/hippodeep)^21^, to derive the eTIV (estimated Total Intracranial Volume).

### Image analysis

**Figure 5:**
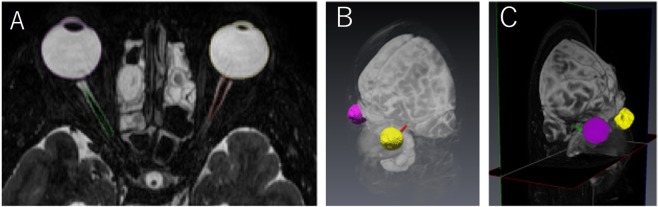
Three-dimensional (3D) eyeball segmentation with Amira. (**A**) A representative axial reconstructed plane of 3D T2-VISTA image. Identical eyes are semi-automatically contoured by threshold method based on their signal intensity. (**B**) 3D volume-rendering reconstructed images of the segmentation of whole eyeballs are derived from image in (**A**). Left anterior oblique view (**B**) and right anterior oblique view (**C**). Yellow and purple segmentations demonstrate volume and shape of the right eyeball and the left eyeball, respectively.

Measurements of overall eye volume and length, including the axial length and transverse length, were conducted by one certificated radiologist (Y.T.) and two trained researchers (A.F. and K.Y.). Images of each eye were separately segmented semi-manually on T2-VISTA multiplanar orthogonal slices, using an image-contour program (Amira 6.4.0, Maxnet Co., Ltd.) (Fig. 5).

**Figure 6:**
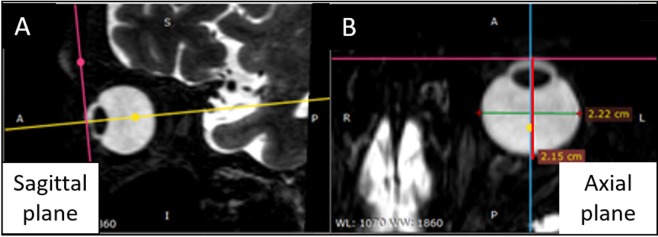
Definition of axial length and transverse length on MRI. (**A**) On the sagittal multi-planar reconstructed plane, the axial plane was defined as the horizontal plane connecting between the top of the cornea and the optic disk. (**B**) On the axial/horizontal plane, the axial length (red line) was defined as the distance between the top of the cornea and the rear part of retina (approximately in the foveal region) in the vertical direction tangential to the corneal curve. The maximum distance orthogonal to the eye axis was defined as the transverse length (green line).

The axial length and transverse length were measured by multi-planar reconstructed view using a DICOM viewer [Radiant DICOM version 4.6.5 (https://www.radiantviewer.com/)]. The axial plane was initially set precisely at the horizontal plane connecting the top of the cornea with the optic disk (Fig. 6). The axial length was then defined as the distance between the top of the cornea and the rear part of the retina (near the fovea) in the vertical direction of the tangent to the corneal curve (eye axis) on this axial plane. Next, the maximum distance oriented orthogonally to the eye axis was defined as the transverse length. To quantify the ellipsoid shapes, the anisotropy ratio was calculated as the axial length divided by the corresponding horizontal length. The higher a ratio is above 1, the more anisotropic (ellipsoid) an eye is in the direction anterior to posterior; a smaller ratio means that the eye is more spherical.

### Creating 3D renderings of average eyes

Using MRI spatial-registration algorithms, we created an “average” eye for each of four groups used to visualize the various morphological characteristics of the eyes: (1) normal/no myopia (controls); (2) normal/myopia; (3) glaucoma/no myopia; (4) glaucoma/myopia. Briefly, an average of all segmented eye masks was created by averaging the eye masks from all participants voxel-wise. Then, each eye mask was spatially aligned on a blurry initial template and were averaged again. Left-eye and right-eye masks were processed independently. Next, the same aligned-averaging process was conducted again, but now within each group, to create group-specific averages. Finally, within each group, the eye masks were smoothed, averaged, and thresholded again, to create the final smooth, group-specific average eye mask.

### Statistical analyses

Data are expressed as the mean ± standard deviation, unless otherwise indicated. To determine how the morphological parameters (including eyeball volume, axial length, transverse length, and anisotropy ratio) and the demographic data (including age, height, eTIV, SE, glaucoma stage, and MD) covaried with each other, Spearman correlation coefficients were calculated using all eye data, including the PPG eyes.

To visualize possible interaction effects between glaucoma and myopia associated with the morphological parameters, an analysis of variance (ANOVA) was performed, followed by a *post-hoc* Tukey test for multiple comparisons. To assess the diagnostic potential of the morphological parameters, we used logistic regression, to discriminate between glaucoma, PPG, and myopia. We evaluated the predictive power of our variables, which included eye-related information (volume, lengths) and subject-related information (age, height, and eTIV), as well as their combinations. We computed receiver operating characteristic (ROC) curves to evaluate the performance of our tests. Despite the relatively large group size of our dataset, we noted and appreciated the large risk of overfitting. Overfitting occurs when a model with many degrees of freedom becomes too well attuned to a specific task during the fitting process. We therefore used a heavy bootstrapping procedure to avoid overfitting. For each diagnostic model that we designed, we generated 200 random splits of our datasets, where one half is used to fit the model parameters, and the other half is used to evaluate the fitted model, such as an ROC curve. This enabled us to generate confidence intervals with each ROC curve. Additionally, only one eye per subject, selected at random, was included in each bootstrap to refrain from overfitting any within-subject correlations.
